# Missed Bilateral Anterior Shoulder Dislocation With Bilateral Coracoid Fracture and Unilateral Long Head of Biceps Rupture

**DOI:** 10.7759/cureus.10996

**Published:** 2020-10-17

**Authors:** Ali H Chamseddine, Mark E Mouchantaf, Kinan Freiha, Ali Asfour, Mohammad O Boushnak

**Affiliations:** 1 Orthopedic Surgery, Faculty of Medical Sciences, Sahel General Hospital, Lebanese University, Beirut, LBN; 2 Orthopedic Surgery, Faculty of Medical Sciences, Lebanese University, Beirut, LBN; 3 Orthopedic Surgery, Faculty of Medical Science, Lebanese University, Beirut, LBN

**Keywords:** lesser tuberosity osteotomy, missed bilateral anterior shoulder dislocation, coracoid process fracture

## Abstract

Missed or chronic bilateral anterior shoulder dislocation is a rare presentation, usually secondary to epileptic attack. We present herein an exceptional case of this injury pattern, associated with bilateral displaced fracture of the coracoid process, and unilateral rupture of the long head of biceps. Treatment consisted of open reduction through osteotomy of the lesser tuberosity, with additional stabilization of the glenohumeral joint, using the Latarjet procedure by transposition of the coracoid fragment with its attached conjoint tendon to the antero-inferior glenoid rim. Rupture of the long head of the biceps required tenodesis. Temporary glenohumeral pin transfixation was performed for residual instability at the end of the procedure. Patients with postictal shoulder pain, discomfort, or disability should be investigated with adequate radiographs, in addition to CT scan or MRI when needed. Early diagnosis allows for safe closed reduction, and helps avoid late and more complex surgical treatment required for missed or chronic dislocations.

## Introduction

Missed bilateral simultaneous anterior shoulder dislocation is a sporadic presentation reported after violent trauma, convulsion, or electrocution [1-7]. We present herein an exceptional case of a similar injury secondary to grand mal seizure in a young adult patient. The injury was undiagnosed for six weeks, and was associated with displaced bilateral coracoid fracture, and unilateral rupture of the long head of biceps. To our knowledge, this association of lesions has never been previously reported. Our aim is to bring attention to this unique association of injuries, especially after an epileptic attack, as well as to discuss modalities of treatment.

## Case presentation

**Figure 1:**
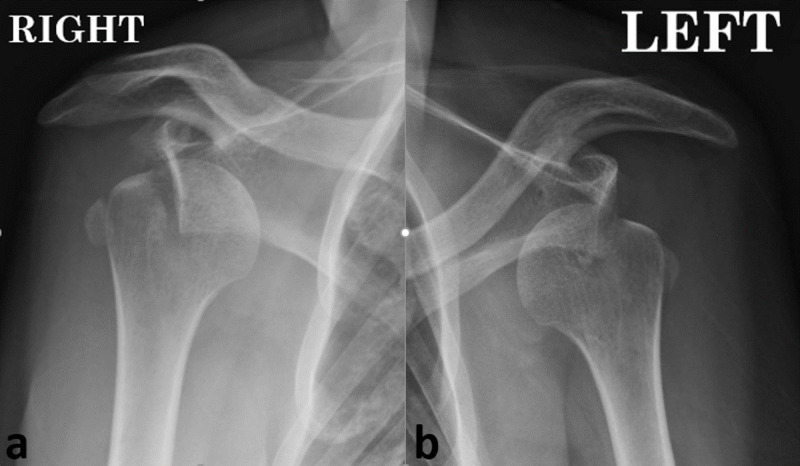
(a, b) Preoperative antero-posterior radiographs

**Figure 2:**
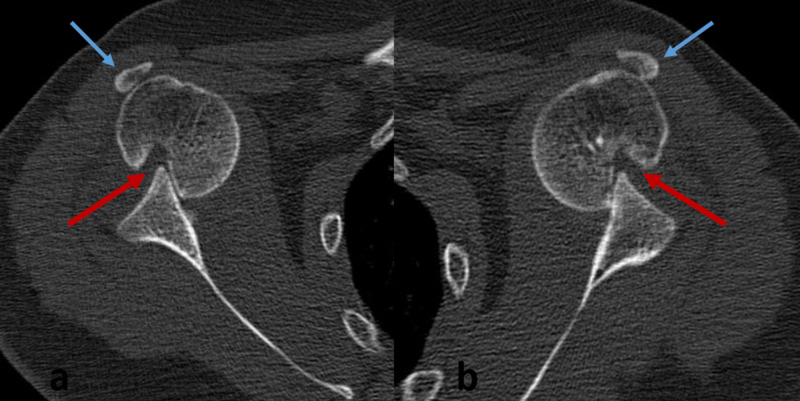
(a, b) CT Scan axial cuts of both shoulders showing bilateral anterior dislocation, with the humeral head locked over the anterior glenoid rim, Hill-Sachs lesion of approximately 20% of the humeral head (red arrow), and fracture of the coracoid tip displaced to the lateral aspect of the greater tuberosity (blue arrow)

A 28-year-old male patient with long history of epilepsy presented for bilateral shoulder pain of six-week duration, associated with very limited range of motion and complete inability to use both upper limbs. The disability occurred after the patient experienced a grand mal epileptic seizure. He was followed up by a neurologist who was mainly focused on regulating his anti-epileptic treatment; the bilateral shoulder complaint was considered as “post-epileptic muscular spasm”. Upon physical examination, both shoulders were locked in mild abduction and external rotation, with notable restriction of range of motion in all directions. Neuro-vascular assessment was bilaterally normal. Radiographs (Figure 1) and CT Scan (Figure 2) of both shoulders revealed bilateral anterior shoulder dislocation, with bilateral displaced fracture of the coracoid tip; the coracoid fragment was displaced to the lateral aspect of the greater tuberosity on both sides. In addition, a Hill-Sachs lesion involving approximately 20% of the humeral head was bilaterally present.

Under general anesthesia, only one gentle attempt for closed reduction using Kocher maneuver was carried out for each shoulder. The closed reduction attempt was unsuccessful on both sides. Surgical reduction was performed for both shoulders within a two-day interval, starting with the right side. Standard deltopectoral approach was used in semi-sitting position. The surgical procedure and findings were identical for both shoulders, except for one additional anatomopathological finding for the left shoulder. The tip of the coracoid process was found avulsed and completely displaced to the lateral aspect of the greater tuberosity, with the conjoint tendon remaining attached to it. The size of the coracoid fragment was estimated at 1.5 cm. The humeral head was locked over the anterior glenoid ridge and could not be reduced.

Osteotomy and elevation of the lesser tuberosity, along with its attached sub-scapularis tendon and capsule, were performed to expose the glenohumeral joint. Articular debridement with removal of fibrous tissue was followed by relocation of the humeral head into the glenoid fossa. Articular inspection revealed an impaction of the posterosuperior aspect of the humeral head as seen on CT Scan, with complete laceration of the anterior glenoid labrum and erosion of the corresponding glenoid rim. The anterior labral remnants were debrided, and the antero-inferior glenoid rim was prepared to receive the coracoid tip, as per the Latarjet procedure. Fixation of the coracoid tip at the antero-inferior glenoid rim was performed using one cancellous screw of 3.5 mm diameter with a washer. The lesser tuberosity was reduced and fixed with two cancellous screws of 3.5 mm diameter with washers.

**Figure 3:**
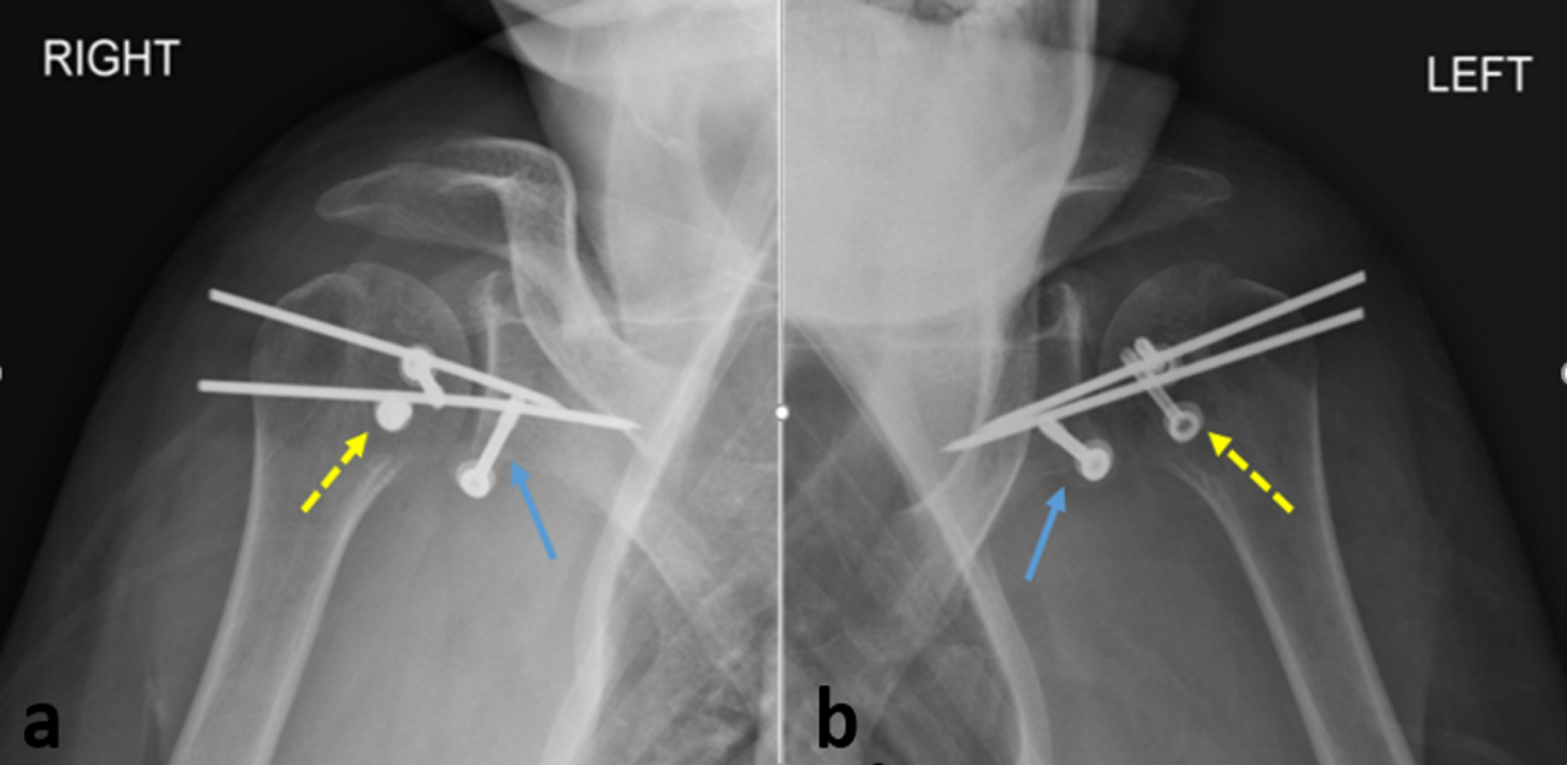
(a, b) Postoperative antero-posterior radiographs of both shoulders showing fixation of the coracoid fragment to the antero-inferior glenoid rim (blue arrow), with fixation of the lesser tuberosity osteotomy (yellow dashed arrow), and pin transfixion of the gleno-humeral joint. Shoulder joint is well reduced in both sides

Shoulder reduction was finally secured with two Kirshner wires of 2.0 mm, inserted from the lateral aspect of the humeral head through the glenoid fossa, due to intra-operative residual glenohumeral instability at the end of the procedure. As additional findings on the left shoulder showed complete rupture of the tendon of the long head of biceps just at the proximal entry of the bicipital groove, tenodesis of the left long head of biceps into the bicipital groove was performed using trans-osseous sutures. Postoperative arm sling was applied on both sides. Radiographic control showed adequate glenohumeral reduction for both shoulders (Figure 3).

**Figure 4:**
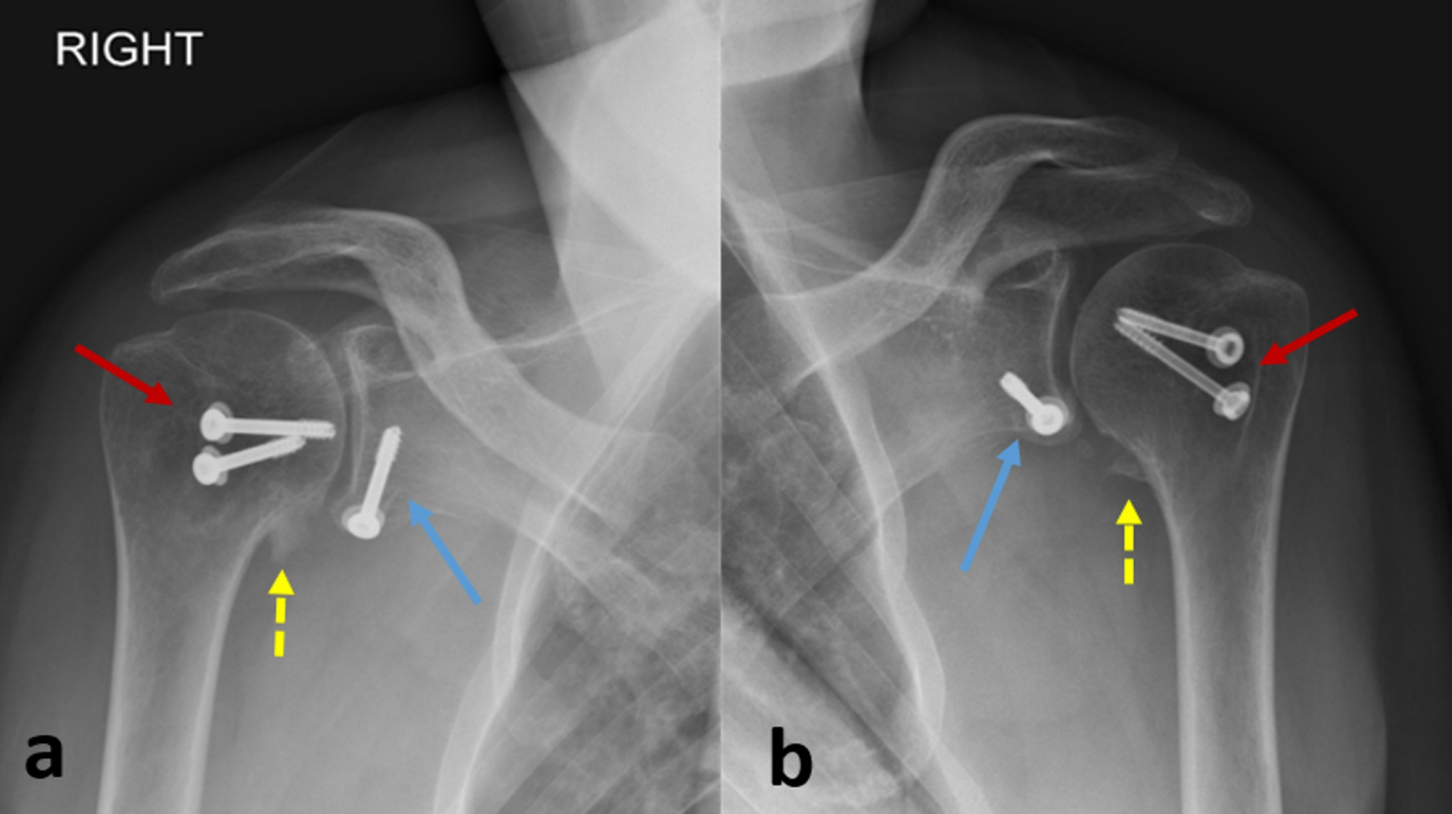
(a, b) Antero-posterior radiographs of both shoulders at 11-month follow-up: complete healing of the coracoid process to the antero-inferior glenoid rim (blue arrow), with concentric reduction of the gleno-humeral joint on both sides. Small ossification is seen at the metaphyso-epiphyseal junction of the proximal humerus bilaterally (yellow dashed arrow). The lesser tuberosity appears well reduced and healed (red arrow)

The patient started an intensive rehabilitation program after removal of the Kirshner wires at four weeks postoperatively; active elbow flexion was prohibited for three months to protect fixation and healing of the transposed coracoid tip. At 11-month follow-up, the patient resumed his profession as a cook; however, he was still on intermittent rehabilitation, and had approximately the same painless active range of motion for both shoulders: 110° forward flexion, 15° extension, 90° abduction, 20° adduction, 30° external rotation, and 40° internal rotation. Radiographic assessment showed concentric glenohumeral reduction, with fusion of the transposed coracoid process to the antero-inferior glenoid rim for both shoulders (Figure 4).

## Discussion

A review of the literature by Ballesteros et al. [1] revealed that missed bilateral anterior shoulder dislocations, spanning more than three weeks’ duration, are almost always secondary to seizure; associated bony lesions were encountered in 47% of cases, and involved fractures of the greater tuberosity and fractures of the proximal humerus in 78% and 22% of cases, respectively. Bilateral coracoid fracture was associated with bilateral anterior shoulder instability in rare occasions [8]. Dunlop [9] noted that 11% of patients with bilateral anterior shoulder dislocation are diagnosed late. It has been stated that bilateral synchronous muscular contraction forces of equivalent intensity during epilepsy can produce bilateral anterior shoulder dislocation [1].

Meanwhile, others believe that seizure attacks produce bilateral posterior rather than bilateral anterior dislocations [3,5]; they suggest that anterior dislocation may be secondary to trauma of the upper limbs hitting the ground, when the patient falls down during the epileptic attack. This explanation remains inconclusive, however, since only one extremity usually receives the energy of the impact when the patient’s body hits the floor. For O'Connor-Read et al. [3], accurate physical examination and adequate radiographic assessment of shoulders are warranted for patients with postictal shoulder pain, discomfort, disability, or deformity. This would avoid late diagnosis, and allow for the implementation of early appropriate treatment in order to improve prognosis. For Saragaglia et al. [10], the most plausible mechanism of associated coracoid fracture, in the context of anterior shoulder dislocation, is the direct impact of the humeral head on the coracoid process. They hypothesized that this mechanism may occur when the coracoid process is lengthier and more laterally oriented than normal, or if the dislocated humeral head is more proximally displaced than usual [10]; however, this mechanism has been contrasted by Ogawa et al. [11]. Another mechanism of coracoid fractures is the traction-avulsion mechanism induced by the powerful traction of the conjoint tendon attached to the coracoid tip, usually without involving anterior shoulder dislocation [11]. According to Saragaglia et al. [10], fractures of the coracoid process can be distinguished into three types: (1) fracture of the base, typically accompanying acromioclavicular dislocations, (2) fracture of the horizontal part, specifically associated with anterior shoulder instability, and (3) fracture-avulsion of the coracoid tip, apparently representing an isolated lesion, induced by powerful contraction of the conjoint tendon. When associated with shoulder dislocation, the coracoid fracture is commonly undisplaced, and the clinical presentation is largely dominated by the dislocation [8,10]. Furthermore, coracoid fractures are usually difficult to visualize on routine radiographs, and are therefore generally initially missed [11]. CT Scan with 3D reconstruction has been recommended for accurate assessment of associated coracoid fracture, as well as helping make accurate therapeutic indication [12]; it significantly helps conclude whether there is sufficient adequate coracoid bone fragment to perform a Latarjet procedure [12].

Closed reduction of chronic unreduced anterior shoulder dislocation is not recommended for injuries of more than six weeks’ duration; it implies high risk of iatrogenic humeral head fracture and neurovascular impairment [13]. In addition, soft tissue interposition and newly formed fibrous tissue in the glenoid fossa, usually interfere with stable reduction. Schulz et al. [13] revealed that functional and subjective results of untreated anterior dislocations can be impressive. They also stated that humeral head excision can be useful for patients with chronic seizure disorders who present missed dislocation associated with humeral head comminution. In contrast, for Flatow et al. [14], untreated patients have notable loss of motion with variable degree of pain; they advocated total shoulder arthroplasty with additional bone graft from the humeral head to reconstruct the anterior glenoid rim defect, when the joint is degenerative. However, we believe the bilateral nature of the injury would preclude the aforementioned therapeutic modalities, e.g. simple observation, resection arthroplasty, and replacement arthroplasty, especially in young patients. Nevertheless, simple observation has been applied by Yadav [6] for missed bilateral anterior dislocation with bilateral greater tuberosity fracture secondary to seizure, in a 56-year-old patient who was able to perform some daily living activities at six-month follow-up, whereas Thomas and Graham [7] performed bilateral hemi-arthroplasty for a patient in the sixth decade. Bilateral closed reduction was possible at seven weeks following injury by Mynter [2]. Mehta et al. [5] successfully performed closed reduction at four weeks for one side in a patient with missed bilateral anterior dislocation, associated with bilateral fracture of the greater tuberosity secondary to seizure; the other side could not be reduced by closed maneuvers and underwent open reduction with capsulorrhaphy, subscapularis repair, and internal fixation of the greater tuberosity. Choulapalle et al. [4] performed open reduction with temporary glenohumeral transfixion, six weeks after the simultaneous bilateral injury. The presence of associated fracture of the coracoid process can be useful when performing the stabilization procedure for recurrent anterior shoulder instability; a large coracoid fragment with intact cortex is usually used to perform the Latarjet procedure [8], whereas a smaller fragment corresponding the coracoid tip may serve to achieve the Boytchev procedure, by rerouting the coracoid tip with its attached conjoint tendon through the distal third or under the subscapularis, before reinserting it back to the stump of the proximal coracoid segment [15,16]. Robinson et al. [12] stated that the method of stabilization is dictated by the location of the coracoid fracture: Latarjet procedure for fractures involving the entire horizontal segment and located at the so-called coracoid elbow, and Eden-Hybinette procedure by transposition of the iliac bone block to the anterior glenoid rim for more distal fractures located at the coracoid tip.

As almost all previous reported cases of missed anterior shoulder dislocation were associated with an intact coracoid process, several authors suggested - after performing glenohumeral open reduction - carrying out an osteotomy of the coracoid process, and using it to achieve either a Latarjet [14,17] or a Boytchev procedure [17]. Protection of the repair with temporary acromiohumeral or glenohumeral pin transfixion has been recommended, particularly in patients with seizure, or whenever stability was in doubt [17-18]. Limited incision of the subscapularis - with preservation of its distal continuity and protection of the anterior circumflex humeral vessels - has been recommended to avoid impairment of humeral head vascularity during open reduction of missed anterior dislocations [17]. Chamseddine et al. [19] reported on the osteotomy of the lesser tuberosity for exposure and reduction of posterior shoulder dislocation; it allows for the preservation of the subscapularis tendon with bone-to-bone healing. We believe this approach is also very useful for open reduction of missed anterior dislocations. Rupture is usually the ultimate sequence of a spectrum of progressive disorders that may affect the long head of biceps in rotator cuff pathology [20]; it remains an exceptional injury pattern in association with missed anterior shoulder dislocation [17]. Many comparative studies have shown similar results for tenodesis and tenotomy of the long head of biceps, when indicated during surgical repair of rotator cuff injuries [20]. Although tenodesis has shown good functional and cosmetic results, the site of tenodesis is controversial, whether proximal, distal, or into the bicipital groove [20].

## Conclusions

In conclusion, patients with any shoulder pain, discomfort, disability or deformity following epileptic attack should be highly suspected for dislocation and investigated accordingly with adequate radiographs and CT scan or MRI. Early diagnosis allows for safe closed reduction and avoids late and more complex surgical treatment. Open reduction of missed anterior dislocation should respect the vascularity of the humeral head; we presume the osteotomy of the lesser tuberosity allows for harmless exposure and safe reduction of the glenohumeral joint. The rupture of the long head of biceps is an exceptional association that requires tenodesis. Associated coracoid fracture can be used to perform either the Latarjet or Boytchev procedure, according to the size of the coracoid fragment and surgeon preference. Finally, temporary glenohumeral or acromiohumeral transfixion is useful to add stability for residually unstable shoulders.
